# Supplementary material for: SPTBN1 Mediates the Cytoplasmic Constraint of PTTG1, Impairing Its Oncogenic Activity in Human Seminoma
Source: Int J Mol Sci. 2023 Nov 29;24(23):16891. doi: 10.3390/ijms242316891 (PMC10707054; doi:10.3390/ijms242316891)
Supplement: Supplementary file 1 [file ijms-24-16891-s001.zip › ijms-2708860-supplementary.pdf]

Table S1. List of triptic unique peptide related to the protein Spectrin alpha chain OS=Homo sapiens; Uniprot code Q13813

Sequence	Modifications	Master Protein Accessions	Theo. MH+ [Da]
QGFVPAAYVK		Q13813	1079,588
LLEATELK		Q13813	916,535
LFGAAEVQR		Q13813	990,5367
LIQSHPESAEDLQEK		Q13813	1723,85
ITALDEFATK		Q13813	1108,588
LSDDNTIGKEEIQQR		Q13813	1745,866
LQTASDESYKDPTNIQSK		Q13813	2024,977
VLETAEDIQER		Q13813	1302,654
YEALKEPMVAR	1xOxidation [M8]	Q13813	1322,677
WSQLLANSAAR		Q13813	1216,643
TATDEAYKDPSNLQGK		Q13813	1737,829
SQLLGSAHEVQR		Q13813	1324,697
SLSAQEEKITALDEFATK		Q13813	1981,012
SLQQLAEER		Q13813	1073,559
SSEEIESAFR		Q13813	1154,532
EKEPIVGSTDYGKDEDSAEALLK		Q13813	2494,22
EKEPIAASTNR	1xGlu->pyro-Glu [N-Term]	Q13813	1197,622
EAFLNTEDKGDSLDSVEALIK		Q13813	2294,14
DVTGAEALLER		Q13813	1173,611
DVDEIEAWISEK		Q13813	1433,679
DLTGVQNLR		Q13813	1015,553
ETENVKSSEEIESAFR		Q13813	1854,872
ELPTAFDYVEFTR		Q13813	1587,769
DLASVQALLR		Q13813	1085,631
DLAALGDKVNSLGETAER		Q13813	1858,951
GRELPTAFDYVEFTR		Q13813	1800,892
HQAFEAELSANQSR		Q13813	1587,751
HQAFEAELHANADR		Q13813	1608,751
KFDDFQK		Q13813	927,4571
KFDDFQKDLK		Q13813	1283,663
FQFFQR		Q13813	872,4414
FLADFR		Q13813	768,4039
FKELSTLRR		Q13813	1149,674
GKDLIGVQNLLK		Q13813	1297,784
GLVSSDELAK		Q13813	1018,542
LIDVNHYAKDEVAAR		Q13813	1713,892
GNAMVEEGHFAAEDVK	1xOxidation [M4]	Q13813	1719,764
GLVSSDELAKDVTGAEALLER		Q13813	2173,135
FLSDFR		Q13813	784,3988
QKLEDSYR	1xGln->pyro-Glu [N-Term]	Q13813	1021,495
LQIASDENYKDPTNLQGK		Q13813	2034,014

Table S2. List of triptic unique peptide related to the protein Spectrin beta chain OS=Homo sapiens; Uniprot code Q01082

Sequence	Modifications	Master Protein Accessions	Theo. MH+ [Da]
LLEVLSGERLPKPTK		Q01082	1680,005
LYAGLKDLAEER		Q01082	1377,737
QALQDTLALYK		Q01082	1263,694
IVSSSDVGHDEYSTQSLVK		Q01082	2050,993
LVSDGNINSDR		Q01082	1189,581
LLDPEDISVDHPDEK		Q01082	1721,823
LFQLNR		Q01082	790,457
ILSSDDYGKDLTSVMR	1xOxidation [M15]	Q01082	1815,879
LTTLELLEVRR		Q01082	1342,805
LTTLELLEVR		Q01082	1186,704
ITDLYTDLRDGR		Q01082	1437,733
LQAAYAGDKADDIQKR		Q01082	1762,908
INAVVETGR		Q01082	958,5316
QLWGLLIEETEKR	1xGln->pyro-Glu [N-Term]	Q01082	1597,858
YKEVAELTR		Q01082	1108,6
VQAVVAVAR		Q01082	912,5625
SLLDACESR	1xCarbamidomethyl [C6]	Q01082	1050,488
SLLDACESRR	1xCarbamidomethyl [C6]	Q01082	1206,59
TALPAQSAATLPAR		Q01082	1367,764
TAGYPNVNIHNFTTSWR		Q01082	1977,957
TAASGIPYHSEVPVSLK		Q01082	1755,928
SQNIVTDSSSLSAEAIR		Q01082	1777,893
ELEAENYHDIKR	1xGlu->pyro-Glu [N-Term]	Q01082	1498,728
EAEKLESEHPDQAQAILSR		Q01082	2151,068
DQNTVETLQR		Q01082	1203,596
DLVAIEAK		Q01082	858,4931
FATDGEGYKPCDPQVIR	1xCarbamidomethyl [C11]	Q01082	1952,917
ETWLSENQR		Q01082	1162,549
EQWANLEQLSAIR		Q01082	1557,802
EIGQSVDEVEK		Q01082	1232,6
AQTLPTSVVTITSESSPGKR		Q01082	2059,103
FATDGEGYKPCDPQVIRDR	1xCarbamidomethyl [C11]	Q01082	2224,045
DALLLWCQMK	1xCarbamidomethyl [C7]; 1xOxidation [M9]	Q01082	1293,633
KHEAIETDIAAYEER		Q01082	1774,861
FMELLEPLNER	1xOxidation [M2]	Q01082	1406,698
FFSMVR	1xOxidation [M4]	Q01082	802,3916
TQILAASYELHK		Q01082	1373,742
KLPEELGRDQNTVETLQR		Q01082	2126,12
LVSQDNFGFDLPAVEAATK		Q01082	2022,018
HRPDLIDFDK		Q01082	1255,643
ALVADSHPESER		Q01082	1310,634
ELALRNELIR		Q01082	1226,722
EVDDLEQWIAER		Q01082	1502,712
DASVAEAWLLGQEPYLSSR		Q01082	2092,035
IQEKVDSIDDR		Q01082	1317,664
MWEVLESTTQTK		Q01082	1452,704
